# HCV full-length genome reconstruction with sequence independent amplification combined with next generation sequencing

**DOI:** 10.1186/1742-4690-9-S1-O6

**Published:** 2012-05-25

**Authors:** Barbara Bartolini, Giombini Emanuela Abbate, Isabella Visco Comandini, Ubaldo D'offizi, Gianpiero Rozera Gabriella Selleri, Marina Ippolito, Giuseppe Capobianchi, Maria Rosaria

**Affiliations:** 1 Virology at National Institute for Infectious Diseases L. Spallanzani, Rome, Italy

## Introduction

HCV genome variability is related to both disease progression and treatment response. De novo high-throughput pyrosequencing was used to obtain full length HCV genome characterization directly from clinical samples.

## Material and methods

Plasma samples from 3 HCV-infected subjects were analyzed (two patients with subtype 1b, one patient with subtype 2a/2c; viral load: 6.0x10^6^,20.8x10^6^ and 7.3x10^6^ IU/ml viral load, respectively). All samples were analyzed in a single run, using sample-specific barcoding adapters. Data were generated with a modified sequence-independent single primer amplification followed by 454 sequencing (GS-FLX Roche, Titanium version), using the shotgun approach. The reads were assembled using cap3 program; HCV contigs were identified using BLAST against full HCV genome database. Reads of HCV contigs were used for genome reconstruction with gs Mapper (Roche software).

## Results

A total of 297,493 reads were obtained (average length 267 bp). Using a minimum read length cut off of 40 nt with >90% identity and >40% overlapping, BLAST analysis classified a total of 27,107 reads (10,682 from patient 1, 11,920 from patient 2, and 4,505 from patient 3) as HCV-specific. In all patients, genome reconstruction was achieved for more than 98 % of the entire HCV genome. The mean coverage was 315, 307 and 142 reads per site for patients 1, 2 and 3, respectively (overall mean coverage: 253 reads per site). Within-patient variability was calculated, resulting in E1 and E2 as the most variable structural genes in all patients, as expected.

## Conclusion

The present study describes a unifying approach for HCV full genome sequencing, based on sequence-independent amplification combined with next generation sequencing. This may represent a relevant innovation, since so-far HCV full genome direct sequencing was based on genotype-specific multiple primer approach and conventional sequencing. The possibility of performing simultaneous analysis of pooled samples may represent a further advantage for cost saving.High coverage allows to analyze virus variability along the entire genome providing important information on possible viral variants which could impact on clinical and therapeutic outcome.

